# Psychological Flexibility Among Competitive Athletes: A Psychometric Investigation of a New Scale

**DOI:** 10.3389/fspor.2020.00110

**Published:** 2020-09-18

**Authors:** Lis Johles, Henrik Gustafsson, Markus Jansson-Fröjmark, Christer Classon, Jon Hasselqvist, Tobias Lundgren

**Affiliations:** ^1^Department of Social and Psychological Studies, Karlstad University, Stockholm, Sweden; ^2^Department of Educational Studies, Karlstad University, Karlstad, Sweden; ^3^Department of Sport and Social Sciences, Norwegian School of Sport Sciences, Oslo, Norway; ^4^Department of Clinical Neuroscience, Centre for Psychiatry Research, Karolinska Institute, Stockholm, Sweden; ^5^Department of Psychology, Stockholm University, Stockholm, Sweden; ^6^Department of Clinical Neuroscience, Centre for Psychiatry Research, Karolinska Institutet, and Stockholm Health Care Services, Stockholm, Sweden

**Keywords:** acceptance, psychometric evaluation, performance, quality of life, psychological flexibility

## Abstract

There is increasing interest in applying acceptance and mindfulness interventions among athletes. However, there is a lack of sport-specific psychometrically evaluated scales to measure the impact of these interventions. The present study describes the development of a measure: the Psychological Flexibility in Sport Scale (PFSS). Its validity was tested in two studies. In the first study, with 152 elite athletes from various sports, explorative factor analysis was used to evaluate the scale's validity, and one factor emerged with seven items. Significant correlations between psychological flexibility, performance, and quality of life were found. Moreover, the PFSS was significantly negatively associated with age, number of years in sport, and number of years as an elite athlete. In the second study, the confirmatory factor analysis with a new population (252 athletes) supported the one-factor solution. Further, positive associations were found with anxiety (BAI) and depression (BDI-I), indicating construct validity. In conclusion, this study presents a scale for measuring psychological flexibility in a broad range of athletes, with satisfactory psychometric properties and the potential to be a useful instrument for both researchers and clinicians in the sport field.

## Introduction

Psychological flexibility is vital in sports as athletes constantly withstand a variety of stressors, anxiety, and pressure when performing. The performance-specific context of sport requires a sustained focus of attention on goal-directed cues, while disengaging from disruptive stimuli (Gardner and Moore, 2007; Moore, 2009). Athletes are expected to have the ability to cope with and regulate their cognitions, emotions, and bodily reactions, and to focus on their performance even in stressful situations (Gardner and Moore, 2004, 2007). Previous research including interventions with mindfulness and acceptance has demonstrated increased psychological flexibility and athletes improving their sport performance (Gross et al., 2018). As internal and external demands and experiences fluctuate, psychological flexibility is important when focusing on performance-relevant cues during training and competition.

Psychological flexibility involves acceptance and the willingness to experience unwanted private events in order to pursue one's values and goals (Hayes et al., 1996, 2006). Researchers in the field of Acceptance and Commitment Therapy (ACT) define psychological flexibility as “the ability to contact the present moment more fully as a conscious human being and to either change or persist when doing so serves valued ends” (Hayes et al., 2006, p. 7). Psychological flexibility originally emerged as a psychological construct, including processes such as acceptance, mindfulness, and values to influence the way clients could relate to anxiety, depression, and stress (Hayes et al., 2006; Brinkborg et al., 2011; A-Tjak et al., 2015). In describing psychological flexibility, Hayes et al. (2006) proposed a model with six core processes: acceptance; contact with the present moment; cognitive defusion; self as a context; committed action; and values within these clinical contexts (Hayes et al., 2006). Acceptance includes taking a stance of non-judgmental awareness and embracing one's cognitions, feelings, and bodily reactions when they occur (Hayes and Strosahl, 2004). Being present in the moment entails a sense of self as a process of ongoing awareness, which can also include exposure to one's cognitions, emotions, and behaviors. However, the purpose of exposure within the framework of ACT is not to lower one's internal reactions but rather to actively try to be in contact with the present moment. In the long run, this may lead to improved vitality and psychological flexibility (Hayes et al., 2004, 2006). Cognitive defusion works by changing, through relational learning, the contexts and functions that take place in specific situations (Hayes et al., 2004); it is a relational, rather than result-oriented, approach to a process (Hayes et al., 2004). Methods include training in defusing from the fusion with one's internal experiences. Self as a context is a core process, which may elevate contact with alternative types of self-experience. Committed action is another core process, which involves defining goals in specific areas along one's valued path and then acting in that valued direction (Hayes et al., 2004). Committed action is closely related to the person's values. In this context, values are defined as chosen qualities of purposive action that can only be instantiated rather than processed as an object (Hayes et al., 2004). Altogether, each core process is related to and interacts with the other processes (Hayes et al., 2004). In short, psychological flexibility is a construct often measured both as an outcome and a process that involves all six core processes intertwined.

The opposite of psychological flexibility is psychological inflexibility, which refers to a rigid dominance of certain unhelpful private events over effective actions, long-term goals, and helpful thoughts and emotions (cf., Bond et al., 2011). Psychological inflexibility often leads to a vicious circle of avoidance of internal experiences and a decrease in psychological flexibility. Athletes who show low psychological flexibility may, just like patients who get stuck in vicious negative avoidance patterns, show less effective behaviors and miss out on opportunities for optimal performance (Moore, 2009). Furthermore, psychological inflexibility is associated with higher symptoms of distress, including anxiety and depression (cf. Ruiz, 2010), something that is also found in athletes (Zhang et al., 2014; Chen et al., 2017). Psychological inflexibility should therefore be associated with higher distress and poor performance.

The Acceptance and Action Questionnaires-II (AAQ-II; Bond et al., 2011), designed to measure experimental avoidance, is one of the most commonly used measures in mindfulness-acceptance-based research (Gross et al., 2018) The AAQ-II scale has also been used in sport settings. For example, it has been used in studies investigating rehabilitation among athletes (Zhang et al., 2014; DeGaetano et al., 2016), and research indicates that the scale can predict psychiatric problems in injured athletes (Baranoff et al., 2015). One needs to be aware of the limitations of the AAQ-II, however, as it has not been adapted to athletic contexts and measures general psychological flexibility, covering multiple aspects of life (Bond et al., 2013). In general, instruments with a better match to the investigated population are preferable (Gregg et al., 2007). In accordance with ACT theory, psychological flexibility might differ in different contexts (Hayes et al., 1999). For example, an athlete could act flexible in a school setting and behave in line with his or her values but have problems in sport due to a fear of failure and adopt rigid behavioral patterns of avoidance. Measures tailored to the specific context are thus considered to have a stronger association with variables measuring people's functioning (Bond et al., 2013). In line with these assumptions, contextual measures of psychological flexibility are commonly adopted, for example acceptance of chronic pain (Mc Cracken et al., 2004), for work settings (Bond et al., 2013) or for diabetes patients (Gregg et al., 2007). Sport settings involve many contextual differences compared with everyday clinical settings. Precise and validated measures are of the utmost importance in attempts to expand our knowledge about the linkage between sport contexts and behavioral change.

The term *psychological flexibility* has been applied within the ACT framework for more than two decades, but there is still a lack of sport-specific measures targeting psychological flexibility in athletes (Bühlmayer et al., 2017; Lundgren et al., 2019). Therefore, the aim of our research was to develop a sport-specific instrument of psychological flexibility and evaluate its initial psychometrical properties. The first goal in Study 1 was to develop a new scale, the Psychological Flexibility in Sport Scale (PFSS), and to examine its psychometric properties. Further, we investigated the scale's respective relationships with performance and with quality of life. In Study 2 we aimed to test the validity of a modified version of the PFSS, and our goal was to confirm the factor structure. We also aimed to investigate the scale's respective relationships with anxiety and with depression in order to further test construct validity.

## Study 1: Development of the PFSS

The purpose of Study 1 was to develop a scale that measures psychological flexibility, to be applied with athletes. We also aimed to investigate the nomological validity by exploring the scale's relationship with quality of life and performance.

## Method

### Inclusion and Exclusion Criteria

Inclusion criteria were active athletes at the sub-elite, elite, and international levels. Participants who were able to understand and speak Swedish were included. Ten athletes who were invited to participate in the study declined. Athletes injured in the past month were excluded due to their inability to complete performance measurements.

### Participants

There were 152 participants (87 females and 63 males, with two participants not stating a gender; mean age 22.3, age range 17–37 years, standard deviation (*SD*) = 4.3. The athletes were recruited from sport associations in Stockholm and three cities in southern Sweden. Athletes from a variety of sports (badminton, handball, soccer, ice hockey, floorball, table tennis, basketball, and tennis) were invited to participate in the study. Overall, 145 participants reported being involved in team sports, six were individual athletes, and one failed to answer the question. Sport-specific variables are found in Table 1.

**Table 1:** Descriptive statistics for the two samples.

		**Sample 1**	**Sample 2**
		**M (SD) or %**	**M (SD) or %**
Age		22.1 (4.2)	17.0 (0.9)
Gender (% female)		54.0%	46.4%
Sport type (%)	Individual	7.6%	18.4%
	Team	92.4%	81.6%
Years practicing the sport		14.8 (4.5)	9.0 (2.7)
Years with current coach		1.6 (2.0)	1.5 (1.8)
Years with current club		4.2 (4.2)	4.1 (4.1)
Years competing at elite level		7.3 (4.2)	2.8 (1.9)
Hours practicing the sport per week		14.3 (5.2)	13.1 (3.3)

## Questionnaires

### Demographic Questionnaire

The demographic questionnaire assessed participants' age, gender, years of athletic experience, and years competing.

### The Brunnsviken Brief Quality of Life Inventory

The Brunnsviken Brief Quality of Life Inventory (BBQ) consists of 12 statements from six life areas. Participants rated the level of importance of these areas as well as their degree of overall life satisfaction. Each statement ranged from 0 (do not agree at all) to 4 (agree completely), generating points ranging between 0 and 96, with higher scores indicating a higher quality of life. The six areas studied were leisure time, view on life, creativity, learning, friends and friendship, and view of self. Examples of questions include “My leisure time is important for my quality of life,” “Being able to be creative is important for my quality of life,” and “I am satisfied with myself as a person. I like and respect myself.” The BBQ showed satisfactory reliability, with good concurrent and convergent validity (Lindner et al., 2016); the Cronbach α reliability for the scale in Lindner's study was 0.76 (Lindner et al., 2016). Based on earlier research (Zhang et al., 2014; Chang et al., 2017), we expected the PFSS to be negatively correlated with quality of life.

### The Borg CR100 Scale

The Borg CR100 scale is a visual evaluation measurement of performance from 0 to 100% (Borg and Borg, 2002), and has been used successfully in similar ways for rating divers' performance (Borg and Love, 2018). The athletes were asked to rate their performance at their most recent match/competition involving world champions in their respective sport, which corresponded to 100 on the scale. The coaches were then asked to answer the same two questions for each athlete. Four performance ratings were collected: two from the athletes and two from the coaches, once and on the same occasion. The athletes were asked to answer two questions that involved rating their performance at their most recent match/competition compared to their personal best, which corresponded to 100 on the scale. Due to the heterogeneous sample of athletes from a range of different sports, the Borg CR100 scale was chosen as the optimal way to measure performance. Based on earlier research and theoretical assumptions (Gardner and Moore, 2004; Bond et al., 2013; Gross et al., 2018; Lundgren et al., 2019), we expected the PFSS to be negatively related to performance.

### Ethics

Data from the survey were collected following approval by the Regional Ethics Board. All participants received both verbal and written information before completing the consent forms, and were informed of the possibility and right to terminate their participation at any time. No compensation was given to the participants or coaches for their participation in the study, and no personal data or details concerning the coaches' identity were collected.

### Procedures

Participants were informed about the study through one e-mail sent to their respective coaches and sports clubs. Hence, all participants were first informed about the study by their coaches, who read our letter to them. A letter explaining the purpose of the study was sent to all coaches. The next step was a follow-up by e-mail or telephone by the researchers in order to schedule a meeting with participants and coaches. Time and place were booked for 45–60 min after a competition, and one or two researchers met with the athletes in a meeting room at this time. The researchers began by introducing themselves and explaining the purpose of the study, and then handed out the consent forms to all athletes. The questionnaires were then given to those who wanted to participate in the study. In addition, the researchers answered questions and, if necessary, instructions were clarified. The survey was conducted by asking the athletes to complete all measurements and include general information such as age, gender when they had reached elite status, amount of training, and whether they were engaging in a team or an individual sport. The coaches were told to go to a separate room to rate the athletes' performance in the same competition the participants rated.

All persons who started the survey completed their participation. No one submitted blank answers after instructions had been given and consent had been provided.

### Development of the PFSS

In order to develop a context-specific measure (see Bond et al., 2013), the PFSS was inspired by the AAQ-II and adapted to the sport context to measure the construct of psychological flexibility and the degree to which a person avoids distressing thoughts, emotions, behaviors, or memories. Similar to the development of the AAQ for diabetes (Gregg et al., 2007) and the AAQ for work settings (Bond et al., 2013), an initial pool of 20 items inspired by the AAQ-II was defined. The items were discussed amongst five researchers, who have expertise in ACT. To increase face validity and conduct an initial feasibility check, the items were sent to an invited group of five elite athletes from different sports (swimming, ice hockey, and soccer), who were asked to answer each item and then participate in a semi-structured interview between the last author and the individual athletes to further understand the feasibility of each item. After the interviews, nine items were chosen to represent aspects of psychological inflexibility, adapted for general sport purposes, that were considered feasible and acceptable by elite athletes. Items are rated on a Likert-type scale ranging from 1 (never true) to 7 (always true).

### Statistical Analysis

The aim was to examine the psychometric properties of the Psychological Flexibility in Sport Scale (PFSS) after the first exploratory factor analysis (EFA). The EFA of Sample 1 was conducted using IBM SPSS Statistics for Windows (Version 22.0. Armonk, NY: IBM Corp.). Before the EFA was conducted, all items were checked with descriptive statistics—e.g., outliers, skewness, and kurtosis—and none of them emerged as problematic. The EFA was used to examine the factor structure of the PFSS, according to recommendations by Costello and Osborne (2005). To examine the factorability of the data, the Kaiser-Meyer-Olkin measure of sampling adequacy (KMO) and the Bartlett Test of Sphericity (BTS) were used. The parallel analysis (O'Connor, 2000) was used to determine the number of factors to retain, as it formally tests the probability that a factor is due to chance and minimizes the over-identification of factors (Wood et al., 2015). Bootstrapping was used when running parallel analysis, with 5,000 cases generated. Factor loadings at a minimum of 0.30, with no or few cross loadings (0.32 or higher) on two or more factors, and communalities > 0.40 were retained (Tabachnik and Fidell, 2001; Costello and Osborne, 2005). Following the recommendations of Costello and Osborne (2005), a factor with five or more strongly loading items (0.50 or more) was regarded as solid, whereas a factor with less than three items was considered weak. Internal consistency of the PFSS was examined using Cronbach's α. Validity was examined through correlations of the PFSS with quality of life domains and performance measures as assessed by the athletes and their coaches.

## Results

### Exploratory Factor Analysis of the PFSS

The preparatory analyses of the factorability of the PFSS demonstrated a KMO index of 0.91 and a significant BTS (χ^2^ = 585.2, *p* < 0.001), which indicated that the data were appropriate for factor analysis. The parallel analysis suggested one factor (O'Connor, 2000), which was further examined according to recommendations (Costello and Osborne, 2005). The first solution, with the inclusion of all nine items, accounted for 49.8% of the variance; however, two items (8 and 9) showed very low communalities (<0.40). Items with low communalities were gradually discarded, which resulted in an increasingly more solid factor structure. In the final solution, which accounted for 61.8% of the variance, items 8 and 9 were removed due to very low communalities. In this final solution with seven items, one factor emerged and was labeled the Psychological Flexibility in Sport Scale (PFSS, Table 2).

**Table 2:** Exploratory factor analysis of the Psychological Flexibility in Sport Scale (PFSS): Final solution with seven items.

**Items**	**Factor loadings**
1. My memories and experiences from previous failures have a negative impact on me when I am performing.	0.76
2. When competing I cannot control my nervousness, and my nervousness affects my performance negatively.	0.65
3. When I am competing my thoughts impair my performance.	0.83
4. When I am competing my feelings impair my performance.	0.71
5. It seems that most athletes can handle their feelings better than I do when they are competing.	0.66
6. Performance anxiety impairs my performance during competitions.	0.82
7. Worry makes my performance worse when I am competing.	0.78

*Items 8 (“I long for the exhilaration I get when I am competing”) and 9 (“When I am thinking of practicing it feels tiresome and this affects my performance during practice”) were excluded in the process of the exploratory factor analysis*.

### The Validity of the PFSS

As shown in Table 3, the PFSS was significantly and negatively associated with age, number of years in one's sport, and number of years as an elite athlete. In other words, high scores on the PFSS were significantly related to low age, fewer years in one's sport, and fewer years as an elite athlete. As shown in Table 4, the PFSS was significantly and negatively associated with four of the quality of life domains: recreation, philosophy of life, creativity, and self-regard. Accordingly, high PFSS scores were significantly associated with low quality of life scores. As shown in Table 5, the PFSS was significantly associated with three performance measures. High PFSS scores were significantly related to low performance scores, as rated by the athletes and their coaches.

**Table 3:** Correlations between PFSS (total with age, gender and parameters related to sports activities.

**Variables**	**1**	**2**	**3**	**4**	**5**	**6**
^1^PFSS						
^2^Age	−0.27^**^					
^3^Gender	0.26^**^	0.13				
^4^Years in sport	−0.27^**^	0.79^**^	0.17			
^5^Years with trainer	0.01	0.24^**^	0.24^**^	0.20^**^		
^6^Years in elite	−0.35^**^	0.72^**^	0.26^**^	0.65^**^	0.23^**^	
^7^Training hours	0.07	0.06	0.17	0.11	0.15	0.18^**^

*For gender*:

1 *Man*,

2 *Woman*.

** *p < 0.01*.

**Table 4:** Correlations between PFSS total with quality of life domains.

**Variables**	**1**	**2**	**3**	**4**	**5**	**6**
^1^PFSS						
^2^Recreation^(a)^	−0.23^**^					
^3^Philosophy of life^(a)^	−0.25^**^	0.41^**^				
^4^Creativity ^(a)^	−0.18^**^	0.37^**^	0.44^**^			
^5^Learning^(a)^	−0.14	0.23^**^	0.31^**^	0.43^**^		
^6^Friends and friendship^(a)^	−0.14	0.20^*^	0.28^**^	0.22^**^	0.24^**^	
^7^Self-regard^(a)^	−0.47^**^	0.32^**^	0.43^**^	0.40^**^	0.17^*^	0.27^**^

(a) *BBQ, Brunnsviken Brief Quality of Life Inventory*.

* *p < 0.05*,

** * < 0.01*.

**Table 5:** Correlations between PFSS total with performance as assessed by the athletes and their trainers.

**Variables**	**1**	**2**	**3**	**4**
^1^PFSS				
^2^Performance: Athlete	−0.26^**^			
^3^Performance: Coach	−0.13	0.25^**^		
^4^Performance vs. world elite: Athlete	−0.24^**^	0.54^**^	0.18^*^	
^5^Performance vs. world elite: Coach	−0.27^**^	0.16	0.60^**^	0.16^*^

* *p < 0.05*,

** *p < 0.01*.

### Study 2: Psychometric Properties of the PFSS

The purpose of the second study was to examine the fit of the one-factor PFSS using confirmatory factor analysis (CFA). In addition, concurrent validity was tested with theoretically expected variables (i.e., anxiety and depression) in order to further validate the scale. Research has shown that experiential avoidance (the opposite of psychological flexibility) is positively related to anxiety and depression (Hayes et al., 2006; Ruiz, 2010; Bond et al., 2011). The PFSS is therefore expected to be negatively related to both depression and anxiety in the same manner.

### Participants

The participants were 252 athletes (121 females and 131 males, with two stating their gender as other; mean age 16.9 years; age range 15–19 years; *SD* = 0.87. Before exclusion, there were 213 basketball players and 48 skiers (see Table 1). One researcher visited the participants at school to explain more about the study. Most participants were competing at a national elite level. A small number of athletes' answers (*n* = 9) were excluded. The most frequent reasons for exclusion were incomplete answers and/or incorrect codes for identification. Sports high school students who were currently active and interested in participating in the study, and who could understand and speak Swedish, were included. Athletes who were not active in their sport or were receiving treatment because of an injury, and those who lacked interest in participating, were excluded from the study. Injured athletes were excluded as some of the questions were related to current performance.

### Procedures

After ethical approval from the Regional Ethics Board, seven high schools that specialized in cross-country skiing and basketball and were enrolled in the Swedish national sports program (NIU) were approached. All coaches at the schools were contacted once by the researchers through e-mail. The schools had been selected through contact with one of the researchers, and a detailed letter explaining the purpose of the study was sent to the schools and coaches who were interested. All coaches informed their students that a researcher had invited all athletes to a meeting, where more detailed information about the study would to be given. The researcher began by introducing herself and explaining the purpose of the study. Consent forms were then given to all the athletes, and were signed and collected on site. Then the questionnaires were given to those who wanted to participate in the study. The questionnaires were answered in a classroom or an auditorium at their schools. The survey was started by asking the athletes to complete all measurements, including general information such as age, gender, when they had reached elite status, amount of training, and whether they were engaging in a team or an individual sport.

## Questionnaires

### Psychological Flexibility in Sport Scale

The modified seven-item version of the PFSS was used in the data collection. The internal consistency of this version was 0.89.

### The Beck Depression Inventory II (BDI–I)

The Swedish version of the revised 21-item Beck Depression Inventory (BDI-I; Beck et al., 2005) was used to measure signs of depression. The BDI-I assesses cognitive and somatic depression, and each item is rated according to the past 2 weeks on a four-point Likert scale from 0 to 3. The score range of the BDI-I is thereby 0–63. The internal consistency Cronbach's alpha is 0.81, and 1-week test-retest reliability is 0.60–0.83 (Beck et al., 1988, 1996). The internal consistency of this study was 0.87.

### The Beck Anxiety Inventory (BAI)

The BAI is a 21-item questionnaire that assesses symptoms of anxiety in the past 7 days. Items are rated according to the past month on a four-point Likert scale from 0 to 3. The score range of the BAI is 0–63. The internal consistency (Cronbach's alpha) is good (0.92), and 1-week test-retest reliability high (0.75; Beck et al., 1988). The Swedish version of the BAI was used (Beck and Steer, 2005), and the internal consistency was found to be 0.90.

### Statistical Analyses

All participant answers were processed using AMOS, an extended version of IBM SPSS Statistics (Version 22.0. Armonk, NY: IBM Corp.). CFA was conducted to investigate factor loadings for the PFSS items and their restrictions, following procedures outlined by Kline (2005). The purpose of a CFA is to hypothetically test the fit of the model (in this case a measurement) with the observed data. The following fit indices were used to determine how well the model fit the data: the chi-square goodness-of-fit index, comparative fit index (CFI), Tucker–Lewis Index (TLI), root-mean-square error of approximation (RMSEA), and standard root-mean-square residual (SRMR). A non-significant chi-square test indicated a good fit, and CFI and TLI above 0.95 suggested an acceptable fit (Kline, 1998a,b; Hu and Bentler, 1999). An RMSEA of 0.06 or less (Hu and Bentler, 1999) and a SRMR of 0.08 or less (Hu and Bentler, 1999) indicated a reasonable error approximation. It is important to note that these fit indices are only guidelines, and should not be interpreted as the golden rules (Marsh et al., 2004). A first-order factor model was tested, and the main items represented their respective latent variables.

## Results

### Confirmatory Factor Analysis of the PFSS

**Figure 1:**
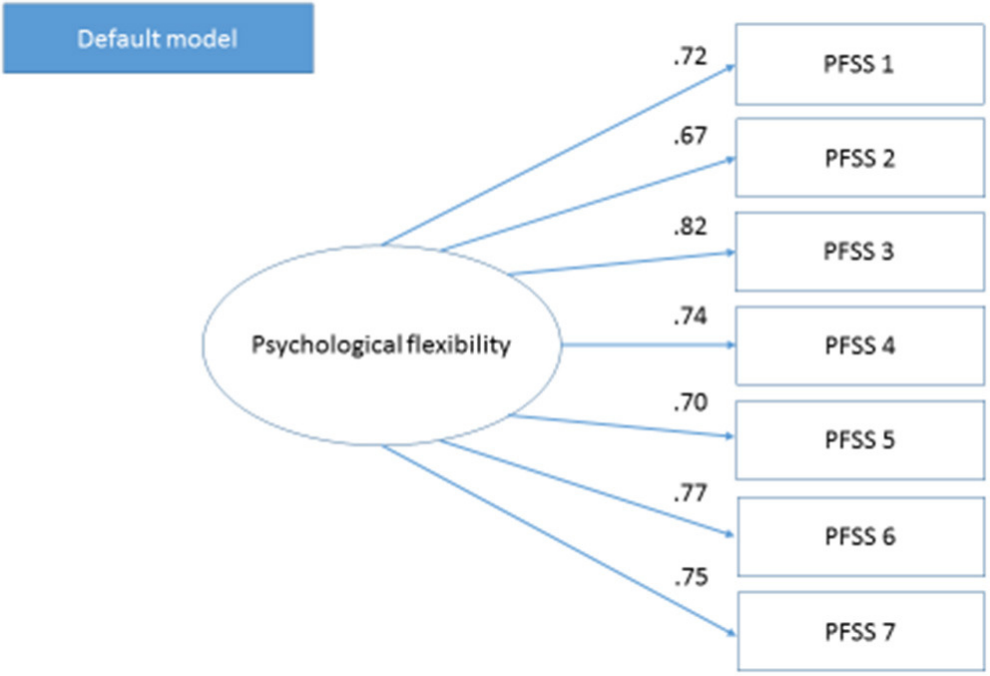
Confirmatory factor analysis of the Psychological Flexibility in Sport Scale in study 2.

The CFA demonstrated an acceptable data fit for the seven-item PFSS. The covariance demonstrated that the chi-square GFI index was χ^2^ = 14.99, df11, *p* = 0.183; CFI was 0.995; TLI was 0.991; RMSEA was 0.038; and SRMR was 0.048. Standardized estimates for the final model are shown in Figure 1. All loadings (i.e., values) of observed variables on the latent variables, correlations, and errors were significant at the *p* > 0.05 level.

### Concurrent Validity

As predicted, we found a significant positive correlation between the PFSS and depression of *r* = 0.49 (*p* > 0.001). Furthermore, the PFSS was also significantly correlated with anxiety, *r* = 0.50, (*p* > 0.001). So higher levels of experiential avoidance were positively associated with higher levels of both depression and anxiety. According to Cohen (1992) recommendations, the strength of these associations was regarded as medium.

## Discussion

The purpose of this study was to develop a measure of psychological flexibility in athletes, the PFSS. Two studies were conducted with two different samples of competitive athletes, providing promising support for adequate structure and internal consistency of this new measure. In the process a pool of items was generated by experts, including both researchers and elite athletes, providing good content validity. Both exploratory and confirmatory factor analysis suggested a one-factor solution for a seven-item scale. The results support the PFSS as a unidimensional measure and a promising tool for measuring psychological flexibility in athletes.

Furthermore, high levels of psychological inflexibility scores were significantly associated with low quality of life, and we also found the PFSS to be positively related to both depression and anxiety. A previous meta-analysis of 27 studies found that psychological flexibility predicted a wide range of quality of life outcomes (Hayes et al., 2006; see also Chawla and Ostafin, 2007), and research using the general AAQ-II has found similar results for psychological flexibility and wellbeing in athletes (Zhang et al., 2014; Chang et al., 2017). The findings in the current study regarding the positive relationship between the PFSS and both anxiety and depression are in line with a substantial amount of research showing similar correlations in a variety of populations (Ruiz, 2010) and in athletes specifically (Zhang et al., 2014; Chang et al., 2017). These findings support the validity of the PFSS.

Moreover, high PFSS scores were significantly related to low performance scores. From an ACT perspective, if athletes can improve their psychological flexibility—that is, reducing experiential avoidance as well as increasing their ability to tolerate unpleasant thoughts and emotions—it would possibly make it easier to pursue value-directed behaviors and focus on task-relevant cues (Moore, 2016). This increases the probability of improving performance (Josefsson et al., 2019). Earlier studies, including MAC interventions, have demonstrated increased psychological flexibility and improved sport performance (Gross et al., 2018; Lundgren et al., 2019). Research in occupational settings has found similar results, indicating that acceptance-predicted mental health and an objective measure of performance including the beneficial effects of having more job control were enhanced when people had higher levels of acceptance (Bond and Bunce, 2003). Hence, our outcomes indicate that athletes could benefit from psychological flexibility in relation to both quality of life, lower levels of distress, and performance.

## Strengths

One strength is that the instrument was developed in close collaboration with elite athletes, and therefore indicates high ecological validity. The population consisted of elite and sub-elite athletes from different sports, which strengthens the studies external validity. The second study confirmed the factor structure of the PFSS and indicated construct validity of the measure. The predicted relationships between the PFSS and the theoretically tied variables of distress, anxiety and depression, as well as quality of life, indicate concurrent and predictive validities.

## Limitations

First, the study was a cross-sectional. Psychological flexibility and the PFSS could merit from being investigated longitudinally, as this flexibility seems to be an ability that evolves gradually over time (Hayes et al., 2006). Future studies focusing on detecting predictors of psychological flexibility across time with various sports and for more than one season could be of great interest. Second, the cultural context and setting in Sweden, with only Swedish-speaking athletes, is a potential limitation that needs further investigation in other cultural contexts. Invariance testing, including both culture and gender, is warranted. Third, the measures of performance were not objectively assessed; while there is promising research on using the Borg CR100 for performance evaluation, objective measures are preferable. Finally, while the measures of depression and anxiety are well validated, we need to further investigate the relationships using sport-specific measures.

## Conclusion and Future Research

In conclusion, this study demonstrates a scale for measuring psychological flexibility in a broad range of athletes, with promising psychometric properties. Psychological flexibility measured with the new PFSS could be a conceivable and applicable complementary psychometrically validated scale in the field of future sport psychology. In the future, the test-retest reliability of the PFSS needs to be evaluated. Moreover, its treatment sensitivity should be investigated in order to determine the potential for using it in evaluating interventions. Most importantly, future research should evaluate the PFSS and examine whether it correlates in the expected direction with established psychology flexibility scales (e.g., AAQ-II) and whether it is potentially more strongly related to sport-specific variables, such as sport anxiety and sport performance, as stipulated by ACT theory (Bond et al., 2013). In addition, we found the PFSS to be more strongly related to negative constructs of wellbeing such as depression and anxiety, and less strongly related to positive psychological constructs (e.g., wellbeing). The association with more positive outcomes need to be investigated in future research.

The present study provides initial evidence that the PFSS has promising psychometric properties. Despite the lack of comparing it with other psychology flexibility scales, it has performed well on the basis of theoretical background (Hayes et al., 2006; Chawla and Ostafin, 2007). The starting point in the present study was to develop a sport-specific measure of psychological flexibility. However, some caution should be used in interpreting the results, and further validation is suggested. Broader perspectives on the relationship between quality of life, burnout, psychological flexibility, and resilience could be of further interest. Further research is needed to examine and refine the psychometric properties of the PFSS; this study marks the beginning of this process.

## Data Availability Statement

The datasets generated for this study are available on request to the corresponding author.

## Ethics Statement

The studies involving human participants were reviewed and approved by Uppsala Regional Ethics Board. Written informed consent from the participants' legal guardian/next of kin was not required to participate in this study in accordance with the national legislation and the institutional requirements.

## Author Contributions

The paper has collectivley developed. Each author contributed to the study planning, data analysis, and interpretation with an additional focus on their respective area of competence. All authors contributed crucially in drafting the aim of the study, concretizing the design, and finishing the manuscript. Inital planning was mainly done by LJ and HG, supported by TL. LJ, CC, and JH was responsible for data collection and initial analysis. MJ-F was essentially responsible for the main statistical analysis, supported by LJ. LJ and HG interpreted the data and together with MJ-F, and wrote the first draft of the manuscript supported by TL. Additionally, LJ managed the communication between all authors during the development of the manuscript. All authors have examined and agreed to the submitted version of the manuscript. HG have assumed responsibility for being the corresponding author, and for keeping co-authors informed of our progress through the editorial review process, the contents of the reviews, and any revisions made.

## Conflict of Interest

The authors declare that the research was conducted in the absence of any commercial or financial relationships that could be construed as a potential conflict of interest.
